# MED14/381: A New Internet Service for a Medical Society and Library (Billrothhaus) in Vienna

**DOI:** 10.2196/jmir.1.suppl1.e57

**Published:** 1999-09-19

**Authors:** M Gschwandtner, CH Muller, H Kritz

**Affiliations:** ^1^Gesellschaft der Ärzte in Wien, ViennaAustria

**Keywords:** Medical Informatics Applications, Information Systems, Online Systems, Databases

## Abstract

Since 1997, the "Gesellschaft der Ärzte in Wien", the oldest medical society in Austria, has been using the possibilities of the Internet to improve the communication with its members. It developed a modern dynamic Web site with different kinds of services: Having one of the largest medical libraries in Austria, it offers remote access to medical databases, full text electronic journals, medical news services like Reuters Medical News and has established a document supply service. Being one of Austria's most traditional platforms where latest developments in medicine are being presented on a very high scientific level, the Internet activities of the society also focus on web-casting projects including live-broadcasting of lectures held in the society as well as on-demand services of these lectures. The modern technological infrastructure built up in the last two years also enables the society to act as an Internet Service Provider for its members and for other medical societies, offering them useful services like unlimited Internet access, Email-accounts, Web space for homepages, virtual servers or server housing. By organising Internet -Workshops for physicians, the society tries to train them how to communicate over the Internet and how to use databases, electronic journals or how to follow online-presentations of congresses and lectures. This paper intends to outline these new possibilities by referring to the Web site of the "Gesellschaft der Ärzte in Wien", making evident how the Internet changes the way medical societies can communicate with their members.

